# Prevalence and Impact of Probable REM Sleep Behavior Disorder in Essential Tremor: A Multicenter Cross‐Sectional Study

**DOI:** 10.1111/ene.70516

**Published:** 2026-02-09

**Authors:** Yuzheng Wang, Mingqiang Li, Runcheng He, Xiaomei Duan, Liang Jin, Dong Chang, JuanWan, Meiqi Jiang, Jiayi Wu, Mingshan Cai, Sheng zeng, Mei Yuan, Heng Wu, Chunyu Wang, Guohua Zhao, Qiying Sun, Beisha Tang, the China Essential Tremor Alliance

**Affiliations:** ^1^ Department of Neurology, Multi‐Omics Research Center for Brain Disorders, The First Affiliated Hospital, Hengyang Medical School University of South China Hengyang Hunan China; ^2^ Department of Neurology, The Second Affiliated Hospital, Hengyang Medical School University of South China Hengyang Hunan China; ^3^ Department of Neurology, The Second Xiangya Hospital Central South University Changsha Hunan China; ^4^ Department of Neurology, The Nanhua Affiliated Hospital, Hengyang Medical School University of South China Hengyang Hunan China; ^5^ Department of Geriatric Neurology, The Second Xiangya Hospital Central South University Changsha Hunan China; ^6^ Department of Neurology, The Fourth Affiliated Hospital, International Institutes of Medicine School of Medicine, Zhejiang University Yiwu Zhejiang China; ^7^ Department of Geriatric Neurology, Xiangya Hospital Central South University Changsha Hunan China; ^8^ Department of Neurology, Xiangya Hospital Central South University Changsha Hunan China

**Keywords:** essential tremor, meta‐analysis, prevalence, rapid eye movement sleep behavior disorder, risk factors

## Abstract

**Objectives:**

To investigate the prevalence of probable REM sleep behavior disorder (pRBD) in essential tremor (ET), identify associated risk factors, and evaluate its effects on motor and non‐motor symptoms.

**Methods:**

Clinical data were collected from 1297 ET patients across multicenter. pRBD was assessed using the RBD Questionnaire‐Hong Kong (RBDQ‐HK). Risk factors associated with pRBD were identified through multivariable logistic regression. Furthermore, a meta‐analysis was conducted to synthesize existing estimates of pRBD/RBD prevalence in ET.

**Results:**

In this study, pRBD was identified in 11.6% of ET patients. Meta‐analysis yielded pooled prevalence estimates of 16% for pRBD (ES = 0.16, 95% CI [0.10–0.21]) and 14% for RBD (ES = 0.14, 95% CI [0.07–0.21]). ET patients with pRBD were older (59.89 ± 13.99 vs. 54.63 ± 16.59 years, *p* < 0.001) and had a later tremor onset (47.55 ± 15.98 vs. 43.15 ± 17.62 years, *p* = 0.007) compared with those without pRBD. ET‐pRBD patients also exhibited a higher frequency of midline tremor (54.67% vs. 43.93%, *p* = 0.003), rest tremor (27.33% vs. 16.04%, *p* = 0.001), and elevated Non‐Motor Symptom Scale (NMSS) scores (20.30 ± 18.50 vs. 10.43 ± 12.42, *p* < 0.001). Multivariable logistic regression identified lower educational attainment (OR = 0.93, *p* = 0.002) and higher NMSS scores (OR = 1.03, *p* < 0.001) as independent risk factors.

**Conclusions:**

pRBD is prevalent in ET and independently associated with lower education and increased non‐motor symptom burden. Recognition of pRBD may help identify an ET subgroup with distinctive clinical features.

## Graphical abstract

In our cohort, probable REM sleep behavior disorder (pRBD) was identified in 11.6% of essential tremor (ET) patients, while a meta‐analysis yielded pooled prevalence estimates of 16% for pRBD and 14% for RBD. Compared to ET patients without pRBD, those patients with pRBD exhibited a higher frequency of midline tremor, rest tremor, and a significantly elevated Non‐Motor Symptoms Scale (NMSS) total score. Multivariable logistic regression identified lower educational attainment and higher NMSS total score as independent risk factors for ET‐pRBD.

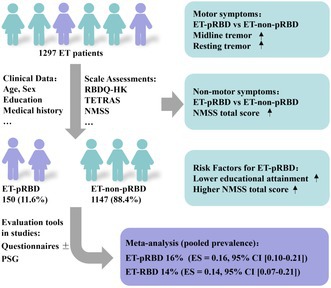

## 1 Introduction

Essential tremor (ET) is a common movement disorder characterized predominantly by a mildly asymmetric action tremor of the upper limbs occurring at a frequency of 4–12 Hz, with or without tremors affecting the head, voice, trunk, and/or lower limbs [1]. The prevalence of ET is approximately 1.33% in the general population, increasing to approximately 5.79% among individuals aged 65 years or older [2]. The etiology and pathogenesis of ET remain incompletely understood, potentially involving environmental factors, aging, and genetic factors [3, 4]. Pathophysiologically, ET is believed to involve rhythmic oscillations within the cerebellum‐thalamus‐cortex circuitry [4].

Clinically, ET is heterogeneous, encompassing both motor and non‐motor symptoms. In addition to classic kinetic tremor, motor manifestations may include soft neurological signs such as resting tremor, impaired tandem gait, questionable dystonic posturing, and questionable myotonia [1, 5]. Non‐motor symptoms also exhibit diverse manifestations, including mild cognitive impairment (MCI) [5], neuropsychiatric disturbances [6], sleep disorders [7, 8], sensory disturbances [9, 10], and autonomic dysfunction [11].

Sleep disturbances are not uncommon in ET and include insomnia, impaired sleep quality, excessive daytime somnolence (EDS), restless legs syndrome (RLS), and rapid eye movement (REM) sleep behavior disorder (RBD) [7, 8]. Emerging evidence indicates a close link between RBD and neurodegenerative disease [12, 13]. Given accumulating data supporting ET as a progressive neurodegenerative disorder [14, 15], clarifying the association between ET and RBD is of considerable clinical and biological interest. However, reported frequencies of probable RBD (pRBD) or RBD in ET vary widely (approximately 1.7% to 43.5%), largely owing to small samples and methodological heterogeneity [7, 8, 16, 17]. Robust estimates from large, well‐characterized cohorts are therefore needed.

To address these gaps, we assembled a multicenter Chinese cohort of 1297 individuals with ET to (i) determine the prevalence of pRBD; (ii) compare motor and non‐motor phenotypes between ET with pRBD (ET‐pRBD) and without pRBD (ET‐npRBD); and (iii) identify factors independently associated with pRBD in ET. In addition, we conducted a meta‐analysis of published studies to estimate the pooled prevalence of pRBD and RBD in ET.

## 2 Methods

### 2.1 Participants

Inpatients and outpatients were recruited from five clinical centers: Xiangya Hospital, Central South University; the Second Xiangya Hospital, Central South University; the First Affiliated Hospital, University of South China; the Second Affiliated Hospital, University of South China; and the Fourth Affiliated Hospital, Zhejiang University School of Medicine, between May 1, 2020 and March 31, 2025. Diagnoses were independently confirmed by at least two experienced neurologists according to the 2018 International Parkinson and Movement Disorder Society diagnostic criteria for ET [1]. Data on sex, age, educational level, age at onset, disease duration, motor and non‐motor symptoms, past medical history, medication use, and sleep patterns were obtained through a detailed interview for all patients with ET. All data were recorded in the Parkinson's Disease & Movement Disorders Multicenter Database and Collaborative Network in China (PD‐MDCNC; http://pd‐mdcnc.com). The study protocol was approved by the Medical Ethics Committee of Xiangya Hospital, Central South University, and by the institutional review boards of all participating centers. All procedures complied with the ethical standards of the Declaration of Helsinki, and written informed consent was obtained from all participants.

### 2.2 Standardized Scale Assessments

Tremor severity was quantified using the Tremor Research Group Essential Tremor Rating Assessment Scale (TETRAS) [5, 18]. Midline tremor was defined as tremor affecting at least one midline structure, such as the neck/head, trunk, face (jaw), tongue, and/or voice [19, 20]. Probable RBD (pRBD) was screened using the validated REM Sleep Behavior Disorder Questionnaire‐Hong Kong (RBDQ‐HK), which comprises 13 items divided into frequency and severity subscales. A total score ≥ 18 or a Part II score ≥ 13 indicated pRBD. The RBDQ‐HK has demonstrated robust reliability and validity in both Chinese and international cohorts [21, 22]. Standardized instructions for the RBDQ‐HK were administered to ensure participant comprehension before responses were recorded. Non‐motor symptoms were assessed with the 30‐item Non‐Motor Symptoms Scale (NMSS), which covers cardiovascular, sleep/fatigue, mood/cognition, perceptual problems, attention/memory, gastrointestinal symptoms, urinary symptoms, sexual function, and other symptoms. Global cognitive status was assessed with the Mini‐Mental State Examination (MMSE).

### 2.3 Statistical Analysis

Data were coded, cleaned, and verified for completeness. Continuous variables were expressed as mean ± standard deviation (SD), and categorical variables were presented as percentages. Categorical data were compared between groups using *χ* ^2^ tests, and continuous variables were analyzed with Mann–Whitney U tests. Multivariable logistic regression was performed to identify risk factors associated with pRBD. To isolate the impact of motor and non‐motor symptoms on pRBD, the model was adjusted for age, sex, age at tremor onset, and education level. All analyses were conducted in SPSS Statistics version 26.0 (IBM Corp, Armonk, NY, USA). Statistical significance was set at *p* < 0.05 (two‐tailed).

### 2.4 Meta‐Analysis

A systematic search of PubMed was conducted on July 31, 2025, using the following Boolean string: ((((((((((Essential Tremor) OR (Essential Tremors)) OR (Tremor, Essential)) OR (Benign Essential Tremor)) OR (Benign Essential Tremors)) OR (Essential Tremors, Benign)) OR (Tremors, Benign Essential)) OR (Familial Tremor)) OR (Tremor, Familial)) OR (Hereditary Essential Tremor)) AND (((((((REM Sleep Behavior Disorder) OR (Behavior Disorder, REM)) OR (Behavior Disorders, REM)) OR (REM Behavior Disorders)) OR (REM Behavior Disorder)) OR (Behavior Disorder, Rapid Eye Movement Sleep)) OR (Rapid Eye Movement Sleep Behavior Disorder)). This query yielded 37 citations. After full‐text review and restricting to studies with sample size ≥ 40, 12 articles were retained. Following exclusion of overlapping publications originating from the same research groups, eight questionnaire‐based pRBD studies (including the present study) and four polysomnography‐confirmed RBD studies were included. Pooled prevalence estimates were calculated using a random‐effects model with 95% confidence intervals.

## 3 Results

### 3.1 Patient Characteristics

A total of 1297 patients with ET were enrolled. Based on the RBDQ‐HK, 150 (11.6%) were classified as ET‐pRBD, and 1147 (88.4%) as ET‐non‐pRBD. Demographic and clinical characteristics are summarized in Table 1. Sex distribution did not significantly differ between ET‐pRBD and ET‐non‐pRBD groups (*p* = 0.554), although ET‐pRBD exhibited a modest female predominance (52.0%). ET‐pRBD patients were significantly older at enrolment (59.89 ± 13.99 vs. 54.63 ± 16.59 years, *p* < 0.001) and had a later age at tremor onset (47.55 ± 15.98 vs. 43.15 ± 17.62 years, *p* = 0.007). Disease duration did not differ significantly between groups (*p* = 0.232). The prevalence of pRBD in ET was positively correlated with age (Figure S1): 3.1% in patients aged < 20 years, increased to 16.59% in those aged 70–79 years, and reached a peak of 18.75% in those aged 80–89 years. ET‐pRBD patients had fewer years of formal education (7.99 ± 4.35 vs. 9.95 ± 4.39, *p* < 0.001) and higher RBDQ‐HK scores (25.86 ± 9.87 vs. 3.90 ± 4.10, p < 0.001). No significant differences were observed between groups regarding body mass index (*p* = 0.602), family history of ET (*p* = 0.170), smoking status (*p* = 0.087), or alcohol consumption (*p* = 0.684).

**TABLE 1:** Baseline demographics and clinical characteristics of the essential tremor cohort by probable rapid eye movement (REM) sleep behavior disorder (pRBD) status.

Items	ET‐pRBD (*n* = 150)	ET‐non‐pRBD (*n* = 1147)	*p*
Male, *n* (%)	72 (48.00)	580 (50.57)	0.554
Age (years)	59.89 ± 13.99	54.63 ± 16.59	**0.000**
Disease duration (years)	12.35 ± 11.03	11.48 ± 10.67	0.232
Age at onset (years)	47.55 ± 15.98	43.15 ± 17.62	**0.007**
BMI	22.79 ± 3.10	22.98 ± 4.20	0.602
Duration of education (years)	7.99 ± 4.35	9.95 ± 4.39	**0.000**
Family history (%)	87 (58.00)	597 (52.05)	0.170
RBDQ‐HK	25.86 ± 9.87	3.90 ± 4.10	**0.000**
Smoking (%)	45 (30.00)	271 (23.63)	0.087
Alcohol consumption (%)	31 (20.67)	221 (19.27)	0.684
Asymmetry of tremor (%)	57 (38.00)	424 (36.96)	0.805
Head (%)	56 (37.33)	372 (32.43)	0.230
Face (%)	44 (29.33)	224 (19.23)	**0.005**
Voice (%)	34 (22.67)	181 (15.78)	**0.033**
Midline tremor (%)	82 (54.67)	481 (43.93)	**0.003**
Resting tremor (%)	41 (27.33)	184 (16.04)	**0.001**
Lower limbs (%)	27 (18.00)	157 (13.68)	0.155
Questionable dystonic posturing (%)	29 (19.33)	182 (15.87)	0.279
Impaired tandem gait (%)	13 (8.67)	101 (8.805)	0.955
Tremor severity			
TETRAS‐I	14.55 ± 9.56	11.99 ± 9.55	**0.001**
TETRAS‐II	18.87 ± 7.60	17.12 ± 7.45	**0.004**
NMSS			
Cardiovascular	0.76 ± 1.70	0.30 ± 0.91	**0.000**
Sleep/fatigue	5.95 ± 6.32	3.10 ± 4.61	**0.000**
Mood/cognition	3.45 ± 6.58	1.75 ± 3.99	**0.000**
Perceptual problems	0.39 ± 1.15	0.13 ± 0.63	**0.000**
Attention/memory	3.43 ± 4.18	1.86 ± 2.89	**0.000**
Gastrointestinal symptoms	1.42 ± 2.71	0.66 ± 1.80	**0.000**
Urinary symptoms	2.96 ± 4.73	1.40 ± 3.11	**0.000**
Sexual function	0.13 ± 1.17	0.03 ± 0.56	0.620
Other symptoms	1.79 ± 2.99	1.19 ± 2.46	**0.001**
Total score	20.30 ± 18.50	10.43 ± 12.42	**0.000**
MMSE	26.83 ± 3.32	27.83 ± 2.70	**0.000**

*Note:* Bold values indicate statistical significance (*p* < 0.05).

Abbreviations: BMI, body mass index; ET, essential tremor; MMSE, Mini‐Mental State Examination; NMSS, Non‐Motor Symptoms Scale; RBD, rapid eye movement (REM) sleep behavior disorder; RBDQ‐HK, REM sleep behavior disorder questionnaire‐Hong Kong; TETRAS, Tremor Research Group Essential Tremor Rating Assessment Scale.

### 3.2 Motor Symptom Characteristics

**FIGURE 1:**
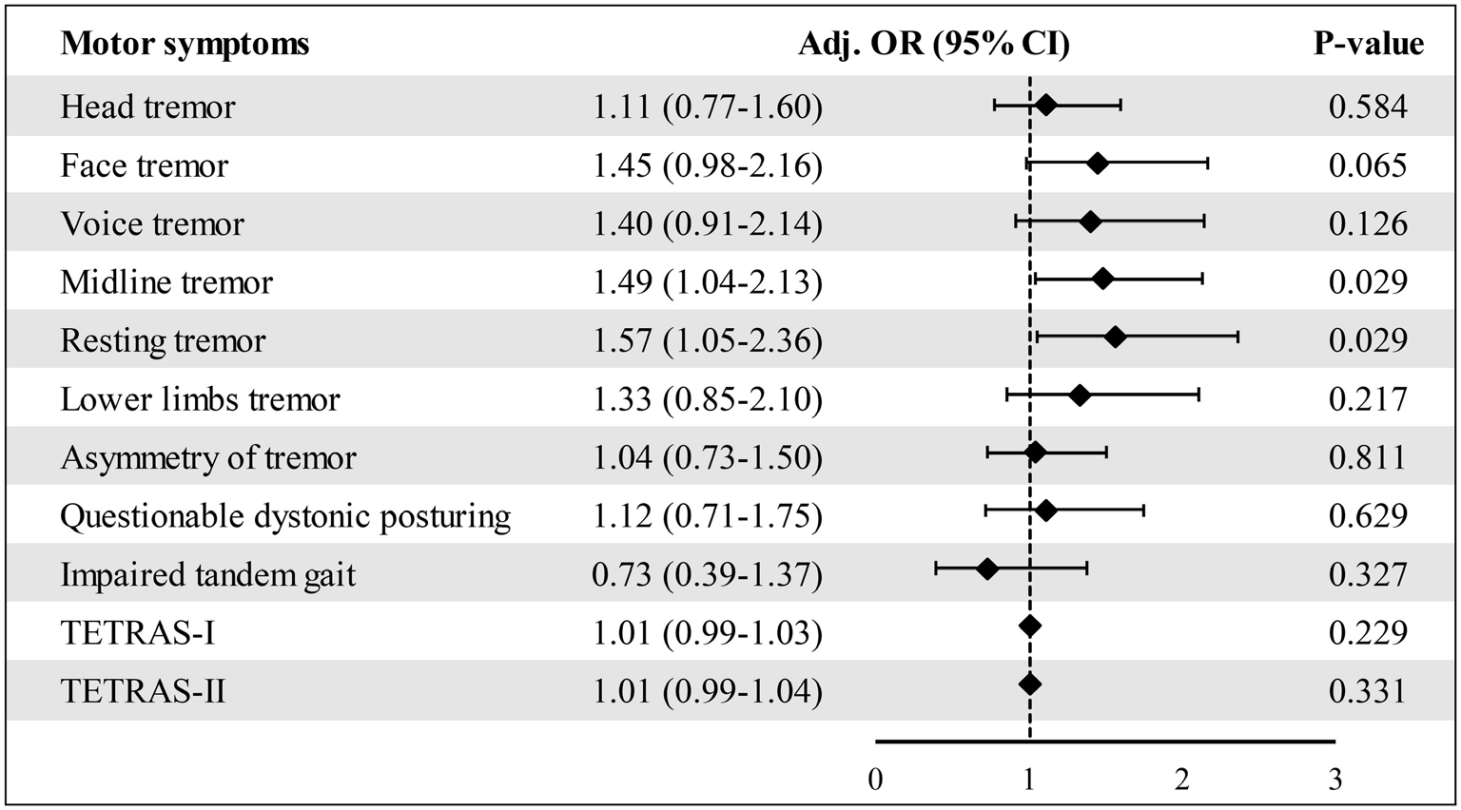
Adjusted associations between pRBD status and motor symptoms in essential tremor. Forest plot comparing ET with pRBD (ET‐pRBD) versus ET without pRBD (ET‐npRBD). Effect sizes are adjusted odds ratios (ORs) with 95% confidence intervals (CIs) from multivariable logistic regression, controlling for age, sex, age at tremor onset (AAO), and years of education. *p* values < 0.05 are considered significant.

As presented in Table 1, midline tremor was more frequent in ET‐pRBD than ET‐non‐pRBD (54.67% vs. 43.93%, *p* = 0.003), primarily driven by increased rates of facial tremor (29.33% vs. 19.23%, *p* = 0.005) and voice tremor (22.67% vs. 15.78%, *p* = 0.033). In contrast, head tremor frequency did not differ between groups (*p* = 0.230). ET‐pRBD patients also exhibited a higher prevalence of rest tremor (27.33% vs. 16.04%, *p* = 0.001). No differences were observed in tremor asymmetry (*p* = 0.805), questionable dystonic posturing (*p* = 0.279), or impaired tandem gait (*p* = 0.955). Both TETRAS‐I (14.55 ± 9.56 vs. 11.99 ± 9.55, *p* = 0.001) and TETRAS‐II (18.87 ± 7.60 vs. 17.12 ± 7.45, *p* = 0.004) scores were significantly higher in ET‐pRBD, indicating greater tremor severity. However, after adjustment for age, sex, age at onset, and education, ET‐pRBD remained significantly associated only with midline tremor (odds ratio [OR] = 1.49, 95% CI: 1.04–2.13, *p* = 0.029) and rest tremor (OR = 1.57, 95% CI: 1.05–2.36, *p* = 0.029), with no significant differences observed in TETRAS‐I (OR = 1.01, 95% CI: 0.99–1.03, *p* = 0.229), TETRAS‐II (OR = 1.01, 95% CI: 0.99–1.04, *p* = 0.331), lower‐limb tremor, questionable dystonic posturing, tandem gait impairment, or other motor symptoms (Figure 1).

### 3.3 Non‐Motor Symptom Characteristics

**FIGURE 2:**
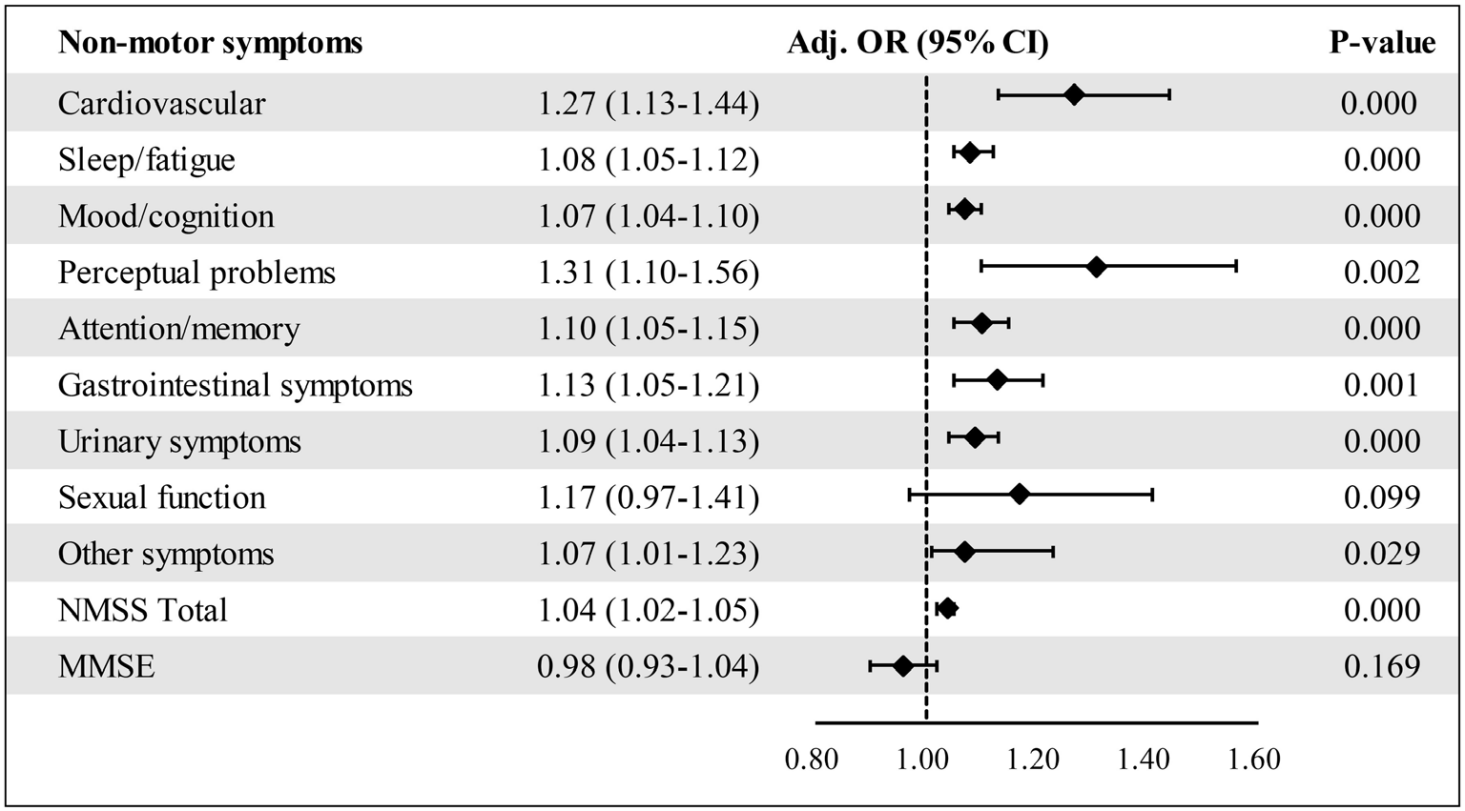
Adjusted associations between pRBD status and non‐motor symptoms. Between‐group comparisons of non‐motor symptom burden in ET‐pRBD vs. ET‐npRBD. The Non‐Motor Symptoms Scale (NMSS) total score is analyzed as a continuous variable; NMSS subdomains are analyzed as present vs. absent; MMSE is analyzed as a continuous variable. Results are shown as adjusted effect sizes with 95% CIs from multivariable models controlling for age, sex, AAO, and years of education (logistic regression for binary subdomains; linear regression for NMSS total and MMSE). *p* values < 0.05 are considered significant.

Total NMSS scores were markedly higher in ET‐pRBD than ET‐non‐pRBD (20.30 ± 18.50 vs. 10.43 ± 12.42, *p* < 0.001), nearly doubling in magnitude (Table 1). All NMSS subdomains, except sexual function (*p* = 0.620), were significantly elevated in ET‐pRBD. ET‐pRBD patients also exhibited lower MMSE scores (26.83 ± 3.32 vs. 27.83 ± 2.70, *p* < 0.001). After adjusting for age, sex, age at onset, and education, sexual dysfunction remained non‐significant (*p* = 0.081), whereas the association with perceptual problems increased (OR = 1.31, 95% CI: 1.10–1.56, *p* = 0.002). The MMSE difference was no longer statistically significant after adjustment (*p* = 0.169) (Figure 2).

### 3.4 Risk Factors for ET‐pRBD

**FIGURE 3:**
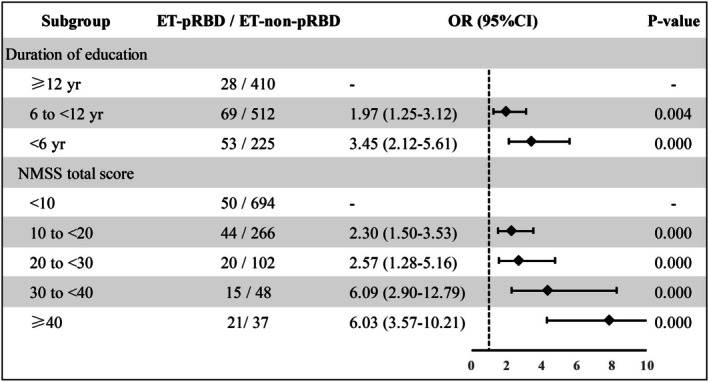
Stratified risk of pRBD by education and NMSS burden. Prevalence of pRBD stratified by years of formal education (> 12, 6 to 12, < 6 years) and by NMSS categories (< 10, 10 to 20, 20 to 30, 30 to 40, > 40). Bars depict point estimates with 95% CIs. *p*‐value for trend is derived from multivariable logistic regression treating education and NMSS categories as ordinal predictors, adjusted for age, sex, and AAO.

In multivariable logistic regression, lower educational attainment (OR = 0.93, *p* = 0.002) and higher total NMSS scores (OR = 1.03, *p* < 0.001) were independent predictors of pRBD (Table 2). Stratification by education level revealed a clear dose–response relationship: pRBD prevalence was 6.39% in those with > 12 years of education, increased to 12.6% in those with 6–12 years (1.97‐fold higher), and peaked at 22.0% in those with < 6 years (3.45‐fold higher) (Figure 3). When stratified by NMSS, the risk of pRBD increased progressively: relative to NMSS < 10, scores of 10–20 and 20–30 were associated with 2.3‐fold and 2.57‐fold higher risk, respectively; scores of 30–40 or > 40 were associated with 6‐fold higher risk (OR = 6.0, 95% CI: 3.8–9.5). Notably, 36.2% of ET patients with NMSS > 40 met pRBD criteria (Figure 3).

**TABLE 2:** Univariable and multivariable logistic regression identifying factors associated with pRBD in essential tremor.

Items	Univariate *p*	Multivariate OR (95% CI)	*p*
Male	0.554	—	—
Age	**0.000**	1.00 (0.98–1.02)	0.958
Age at onset	**0.004**	1.00 (0.99–1.02)	0.645
Disease duration	0.351	—	—
Duration of education	**0.000**	0.93 (0.89–0.98)	**0.005**
BMI	0.599	—	—
Asymmetry of tremor	0.805	—	—
Midline tremor	**0.003**	1.28 (0.88–1.86)	0.203
Head	0.231	—	—
Face	0.006	—	Not included
Voice	0.034	—	Not included
Resting tremor	**0.001**	1.35 (0.88–2.06)	0.170
Lower limbs	0.156	—	—
TETRAS‐I	**0.002**	1.00 (0.97–1.03)	0.950
TETRAS‐II	**0.007**	0.99 (0.96–1.04)	0.808
NMSS	**0.000**	1.03 (1.02–1.04)	**0.000**
MMSE	**0.000**	1.00 (0.93–1.07)	0.960

*Note:* Bold values indicate statistical significance (*p* < 0.05).

Abbreviations: BMI, body mass index; MMSE, Mini‐Mental State Examination; NMSS, Non‐Motor Symptoms Scale; TETRAS, Tremor Research Group Essential Tremor Rating Assessment Scale.

### 3.5 Meta‐Analysis

**FIGURE 4:**
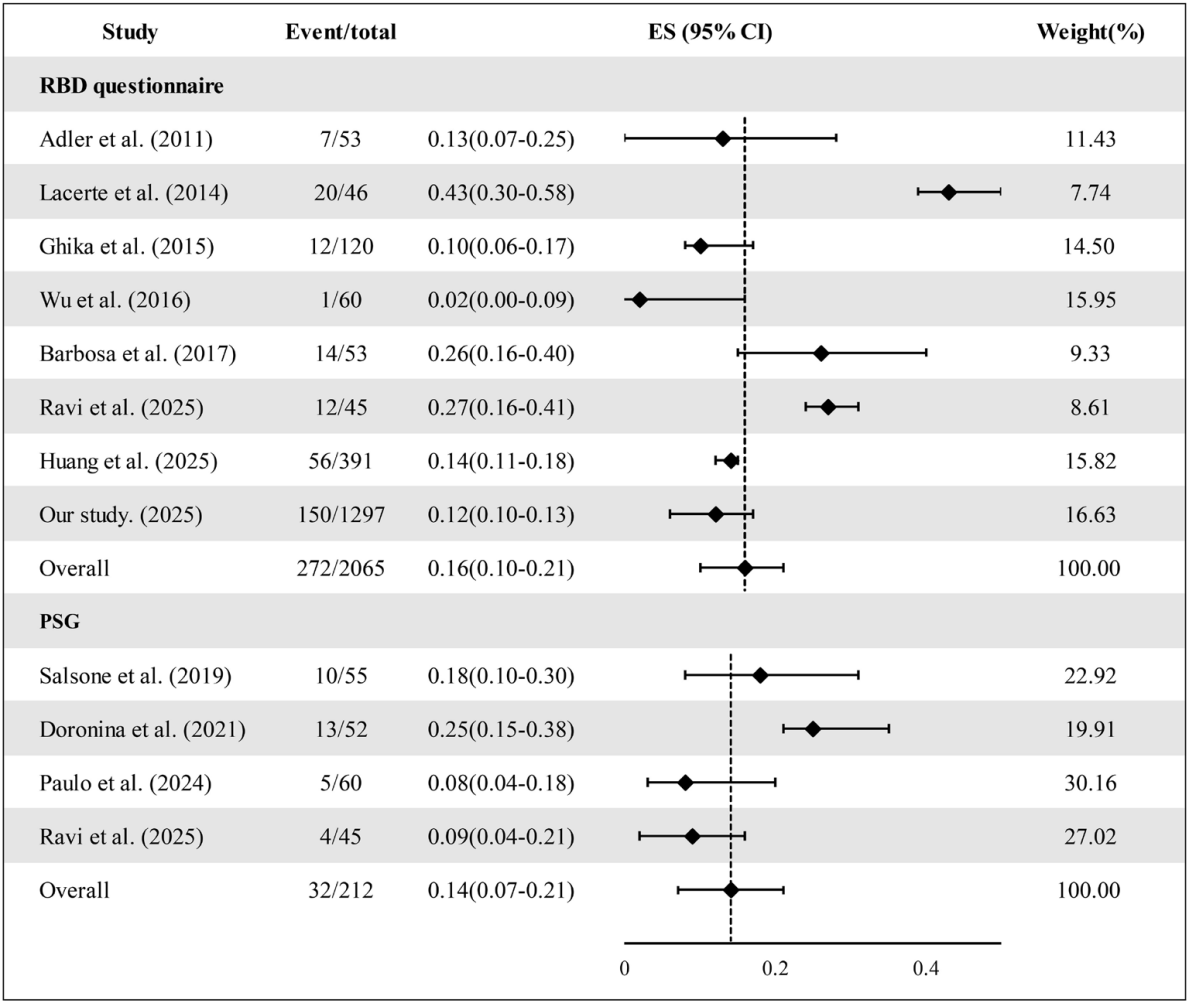
Meta‐analysis of pRBD and RBD prevalence in essential tremor. Random‐effects (DerSimonian–Laird) meta‐analysis of questionnaire‐based pRBD studies and polysomnography‐confirmed RBD studies. Forest plots depict study‐specific prevalence (effect size) with 95% CIs and the pooled estimate; heterogeneity is summarized with *I* ^2^ and Cochran's *Q* statistics.

Across eight questionnaire‐based studies, the pooled prevalence of pRBD was 16% (ES 95% CI = 0.16 [0.10–0.21], *I* ^2^ = 89.71%, *p* < 0.01). Four polysomnography‐confirmed studies yielded a pooled prevalence of definite RBD of 14% (ES 95% CI = 0.14 [0.07–0.21], *I* ^2^ = 60.58%, *p* = 0.05) (Figure 4).

## 4 Discussion

In this multicenter cohort, 11.6% of patients with ET met questionnaire‐based criteria for probable RBD (pRBD). Compared with those without pRBD, affected individuals were older, had a later tremor onset, fewer years of formal education, a higher prevalence of axial and rest tremor, and a substantially greater burden of non‐motor symptoms. Lower educational attainment and higher NMSS scores independently predicted pRBD. To our knowledge, this is the largest multicenter cohort study to date focused specifically on ET‐pRBD, and it broadens the clinical spectrum of sleep disturbance in ET.

At present, ET‐pRBD is mainly ascertained using validated questionnaires such as the Mayo Sleep Questionnaire (MSQ), the REM sleep behavior disorder screening questionnaire (RBDSQ), and the RBD Single Question (RBD1Q), while polysomnography (PSG) is employed in selected centers for definitive diagnosis. Reported frequencies of pRBD in ET cohorts range from 1.7% to 43.5%, and rise to 51.9% in cohorts of essential tremor‐Parkinson's disease (ET‐PD) (Table S1). This broad variability likely reflects differences in targeted patient populations, diagnostic criteria, and assessment methods. Using the RBDQ‐HK, we observed an 11.6% prevalence of ET‐pRBD, which is consistent with the 14.3% reported by Hongyan Huang's group in China using the RBDSQ [16, 23]. Meta‐analyses yielded pooled prevalence estimates of 16% for pRBD (*I* ^2^ = 89.71%, *p* < 0.01) and 14% for PSG‐confirmed RBD (*I* ^2^ = 60.58%, *p* = 0.05), indicating pronounced heterogeneity among studies using RBD‐related scales and a more reliable estimate for PSG‐confirmed RBD, which indirectly validates the findings from our cohort. Bugalho et al. reported PSG‐confirmed ET‐RBD prevalences of 11.6% (2021) and 8.3% (2024) in their studies, which first screened for pRBD via questionnaire [24, 25]. This variation highlights significant heterogeneity in ET‐RBD prevalence across different timeframes and partially different populations.

In previous validation studies against PSG‐based diagnosis, the RBDQ‐HK has shown good performance in identifying RBD, with a sensitivity of 82.2%~85.0%, specificity of 81.0%~86.9%, AUC of 0.89%~0.90, and a Cronbach's alpha of 0.9, indicating acceptable internal consistency and diagnostic accuracy when evaluated against PSG [21, 22]. However, it is primarily designed as a screening tool, and definitive diagnosis still requires PSG. In our study, the RBDQ‐HK was used to screen ET patients for pRBD without PSG confirmation; thus, our estimate reflects the prevalence of ET‐pRBD rather than PSG‐confirmed ET‐RBD. Typically, questionnaire‐based pRBD prevalence exceeds that confirmed by PSG. In our cohort, the observed pRBD prevalence was 11.6%, which is already close to the pooled ET‐RBD prevalence of 14% from our meta‐analysis. This suggests that the true ET‐RBD prevalence in this population is lower than this meta‐analytic estimate and some previous reports. Prospective studies incorporating PSG in Chinese ET cohorts are warranted to confirm these findings.

The reported prevalence of RBD ranges from 0.25% to 1.15% in the general population [26, 27, 28], 23.6% to 46% in Parkinson's disease(PD) [29, 30], and 1.7% to 43.5% in ET, suggesting that ET‐RBD occupies an intermediate position between healthy controls and PD populations. This trend in RBD prevalence parallels the broader profile of non‐motor symptoms in ET, which is also intermediate between that seen in healthy aging and PD [31]. Notably, Bugalho et al. reported that ET patients with concomitant RBD have a higher probability of prodromal Parkinson's disease (PPD) [25]. This finding highlights the importance of monitoring ET‐pRBD patients to enable early identification of signs of conversion to PD.

ET‐pRBD was associated with more prominent midline tremor and rest tremor, as well as a tendency toward higher TETRAS scores. These observations agree with Bugalho et al. [24], who reported universal rest tremor and higher (albeit non‐significant) TETRAS scores in ET‐pRBD. Beyond motor manifestations, our data show a markedly greater non‐motor symptoms (NMS) burden in ET‐pRBD. Prior studies have linked ET‐pRBD to cognitive impairment [32] and to more severe autonomic dysfunction [33]. Analogous patterns are well described in PD, where pRBD tracks with greater NMS burden [34], specific sensory phenotypes such as hyposmia [35], and overall more severe motor and non‐motor profiles than in PD without RBD [36, 37].

We identified two independent correlates of ET‐pRBD: lower education and higher NMSS scores. The education effect aligns with reports across diverse cohorts [38, 39] and may reflect cognitive reserve—whereby higher educational attainment mitigates clinical expression of underlying pathology [40]. Speculatively, reserve‐related modulation of locus coeruleus–noradrenergic (LC/NA) tone might also influence RBD risk [30]. Cognitive impairment, sensory disturbances, and NMS, as common manifestations of neurodegenerative diseases, are considered to possibly precede or parallel with the motor symptoms of ET [41, 42]. Studies on other NMS and RBD in ET are limited; Barbosa et al. [33] have demonstrated an association between RBD and autonomic dysfunction in ET. While the mechanisms by which NMS exacerbate or contribute to RBD remain uncertain, it is hypothesized that the close anatomical proximity of the neural substrates mediating certain NMS to those involved in RBD may contribute to the emergence of RBD in neurodegenerative conditions [43].

Our study has several limitations. First, the questionnaire‐based assessment of ET‐pRBD in our study is subject to potential bias arising from differences in educational attainment, particularly in participants with lower education levels. Consequently, confirmation of our findings by PSG in future research is needed. Second, due to its cross‐sectional design, our study cannot reflect disease progression and outcomes of ET‐pRBD patients over time. Therefore, future longitudinal follow‐up studies employing PSG confirmation among ET patients with probable RBD will be necessary to observe and clarify disease evolution and definitive clinical outcomes.

## 5 Conclusions

Our study demonstrates that pRBD (RBD) is prevalent among patients with ET and is independently associated with lower educational attainment and a greater burden of non‐motor symptoms. The recognition of pRBD (RBD) is therefore critical, as it helps to identify a distinct ET subgroup with unique clinical features.

## Author Contributions

All authors contributed to the study conception and design. Material preparation and data collection were carried out by Y.W., M.L., R.H., X.D., L.J., D.C., J.W., M.J., J.W., M.C., S.Z., M.Y., H.W., C.W., G.Z., Q.S., and B.T. Data analysis was conducted by Y.W., M.L., R.H., and Q.S. The first draft of the manuscript was written by Y.W., Q.S., and B.T. All authors reviewed and edited previous versions of the manuscript and read and approved the final manuscript.

## Conflicts of Interest

The authors declare no conflicts of interest.

## Supporting information


**Figure S1:** A positive correlation between age and the prevalence of pRBD in ET.

**Table S1:** Systematic review of studies reporting the prevalence of questionnaire‐based pRBD and polysomnography‐confirmed RBD in essential tremor.

## Data Availability

The data for this study may be shared upon reasonable request to the corresponding author.
