# Supplementary figures and images for: Prevalence and Impact of Probable REM Sleep Behavior Disorder in Essential Tremor: A Multicenter Cross‐Sectional Study
Source: Eur J Neurol. 2026 Feb 9;33(2):e70516. doi: 10.1111/ene.70516 (PMC12886745; doi:10.1111/ene.70516)

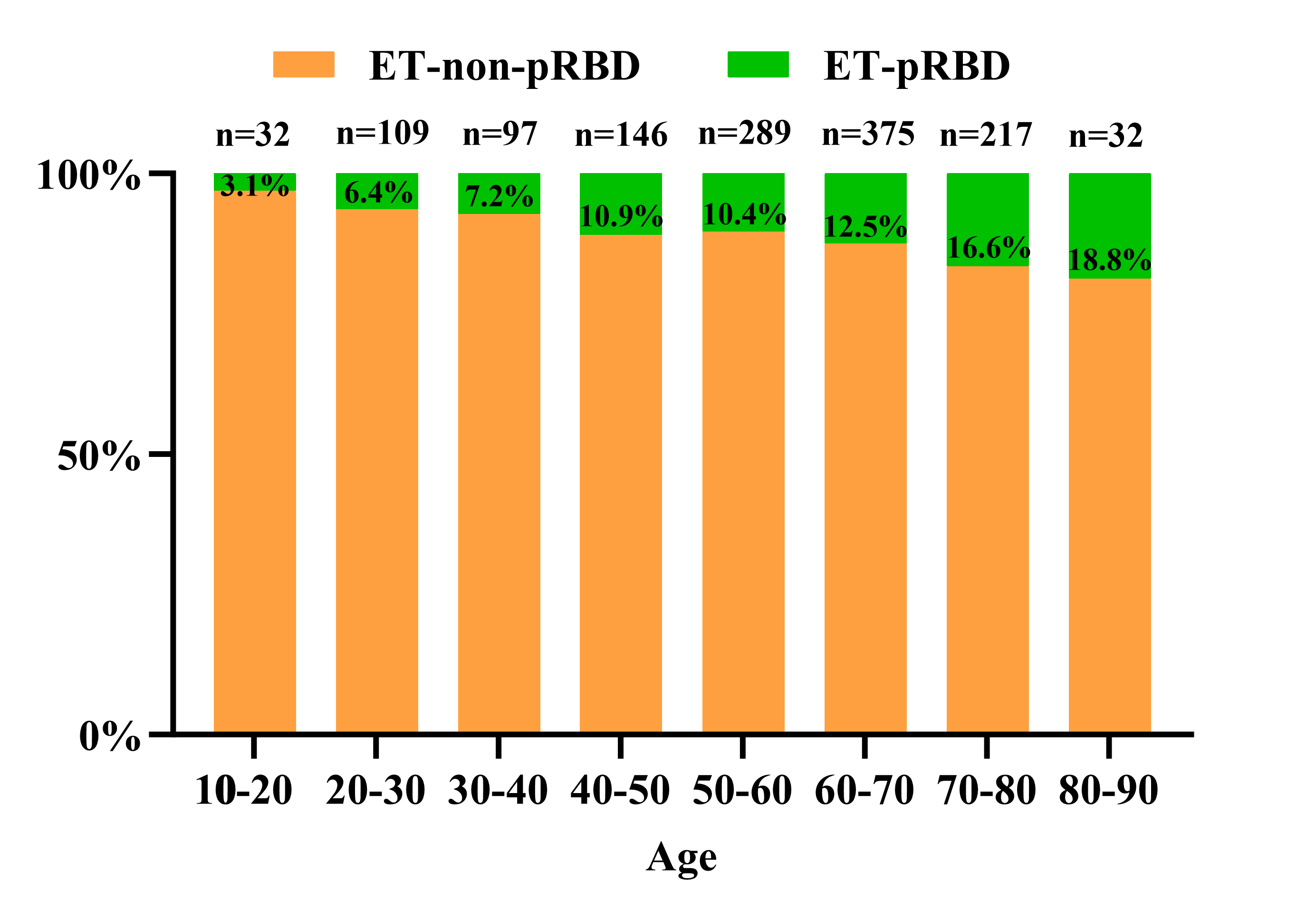

Supplement: Supplementary file 1 — Figure S1: A positive correlation between age and the prevalence of pRBD in ET. [file ENE-33-e70516-s002.tif]
