# Supplementary material for: Prevalence and Impact of Probable REM Sleep Behavior Disorder in Essential Tremor: A Multicenter Cross‐Sectional Study
Source: Eur J Neurol. 2026 Feb 9;33(2):e70516. doi: 10.1111/ene.70516 (PMC12886745; doi:10.1111/ene.70516)
Supplement: Supplementary file 2 — Table S1: Systematic review of studies reporting the prevalence of questionnaire‐based pRBD and polysomnography‐confirmed RBD in essential tremor. [file ENE-33-e70516-s001.docx]

**Supplementary Table 1** Systematic review of studies reporting the prevalence of questionnaire-based pRBD and polysomnography-confirmed RBD in essential tremor.

| Study | County | RBD events / total | Percentage of patents with concomitant RBD | Mean age of RBD (SD) | Mean disease duration of RBD (SD) | Percentage of males in RBD | Method of RBD diagnosis |
| --- | --- | --- | --- | --- | --- | --- | --- |
| Our study | China | 150 / 1297 ET | 11.6% | 59.89(13.99) | 12.35 (11.04) | 48.00% | RBDQ-HK |
| Adler et al., 2011[1] | USA | 7 / 53 ET | 13% |  |  |  | MSQ |
| Giorelli et al., 2014[2] | Italy | 6/22 ET | 27.3% |  |  |  | Questionnaire |
| Lacerte et al., 2014[3] | Canada | 20/46 ET | 43.5% |  |  | 30.0% | RBDSQ |
| Ghika et al., 2015[4] | Greece | 12/120 ET  28/54 ET-PD | 10%  51.9% |  |  |  | Questionnaire |
| Kwon et al., 2016[5] | South Korea | 3/20 ET | 15% |  |  |  | Questionnaire |
| Wu et al., 2016[6] | China | 1/60 ET | 1.7% |  |  |  | RBDSQ |
| Barbosa et al., 2017[7] | Portugal | 14/53 ET | 26.4% | 70.1 (15.5) | 18.3 (15.8) | 42.9% | RBDSQ |
| Ryu et al., 2017[8] | South Korea | 7/25 ET-PD | 28% |  |  |  | RBDSQ+MSQ1 |
| Salsone et al., 2019[9] | Italy | 10/55 ET | 18.2% | 62.9 (12.2) | 10.1 (9.2) | 70.0% | RBD1Q +PSG |
| Huang et al., 2020[10] | China | 34 / 238 ET  10 / 121 pure ET  24 / 117 ET-plus | 14.3%  8.3%  20.5% |  |  |  | RBDSQ |
| Lau et al., 2020[11] | USA | 1 / 33 ET  13 / 33 ET-plus  12 / 35 ET-PD | 3%  38%  35% |  |  |  | Questionnaire |
| Bugalho et al., 2021[12] | Portugal | 5 / 43 ET  13 / 49 ET | 11.6%  23.5% | 76.3 (6.13) | 8.0 (2.16) | 40.0% | PSG  RBDSQ |
| Doronina et al., 2021[13] | Russia | 13 / 52 ET | 25% |  |  | 53.8% | PSG |
| Paulo Bugalho.,2024[14] | Portugal | 5/60 ET | 8.3% | 74.6(3.2) | 12.0(4.5) | 60.0% | RBDSQ+PSG |
| Ravi Prakash Singh., 2025 [15] | India | 12/45 ET  4/45 ET | 26.7%  8.9% |  |  |  | MSQ  PSG |
| Huang et al., 2025 [16] | China | 56/391 ET | 14.3% | 61.43 (13.02) | 12.09 (10.72) | 60.7% | RBDSQ |

ET, essential tremor; RBD, rapid eye movement (REM) sleep behavior disorder; RBDQ-HK, REM sleep behaviour disorder questionnaire-Hong Kong;MSQ, Mayo Sleep Questionnaire; PD, Parkinson’s disease; RBDSQ, RBD Screening Questionnaire; RBD1Q, RBD Single Question; PSG, polysomnography

1. Adler CH, Hentz JG, Shill HA, et al. Probable RBD is increased in Parkinson's disease but not in essential tremor or restless legs syndrome. Parkinsonism Relat Disord 2011;**17**(6):456-8 doi: 10.1016/j.parkreldis.2011.03.007 [published Online First: 20110408].

2. Giorelli M, Bagnoli J, Consiglio L, et al. Do non-motor symptoms in Parkinson's disease differ from essential tremor before initial diagnosis? A clinical and scintigraphic study. Parkinsonism Relat Disord 2014;20(1):17-21 doi: 10.1016/j.parkreldis.2013.09.004 [published Online First: 20130912].

3. Lacerte A, Chouinard S, Jodoin N, Bernard G, Rouleau GA, Panisset M. Increased Prevalence of Non-motor Symptoms in Essential Tremor. Tremor Other Hyperkinet Mov (N Y) 2014;4:162 doi: 10.7916/d82v2d91 [published Online First: 20140902].

4. Ghika A, Kyrozis A, Potagas C, Louis ED. Motor and Non-motor Features: Differences between Patients with Isolated Essential Tremor and Patients with Both Essential Tremor and Parkinson's Disease. Tremor Other Hyperkinet Mov (N Y) 2015;5:335 doi: 10.7916/d83777wk [published Online First: 20150814].

5. Kwon KY, Lee HM, Lee SM, Kang SH, Koh SB. Comparison of motor and non-motor features between essential tremor and tremor dominant Parkinson's disease. J Neurol Sci 2016;361:34-8 doi: 10.1016/j.jns.2015.12.016 [published Online First: 20151210].

6. Wu Y, Wang X, Wang C, et al. Prevalence and clinical features of non-motor symptoms of essential tremor in Shanghai rural area. Parkinsonism Relat Disord 2016;22:15-20 doi: 10.1016/j.parkreldis.2015.10.617.

7. Barbosa R, Mendonça M, Ladeira F, Miguel R, Bugalho P. Probable REM-Sleep Behavior Disorder and Dysautonomic Symptoms in Essential Tremor. Tremor Other Hyperkinet Mov (N Y) 2017;7:522 doi: 10.7916/d8z61vw5 [published Online First: 20171229].

8. Ryu DW, Lee SH, Oh YS, et al. Clinical Characteristics of Parkinson's Disease Developed from Essential Tremor. J Parkinsons Dis 2017;7(2):369-76 doi: 10.3233/jpd-160992.

9. Salsone M, Arabia G, Manfredini L, et al. REM-Sleep Behavior Disorder in Patients With Essential Tremor: What Is Its Clinical Significance? Front Neurol 2019;10:315 doi: 10.3389/fneur.2019.00315 [published Online First: 20190424].

10. Huang H, Yang X, Zhao Q, et al. Clinical characteristics of patients with essential tremor or essential tremor plus. Acta Neurol Scand 2020;141(4):335-41 doi: 10.1111/ane.13209 [published Online First: 20200106].

11. Lau HL, Marmol SE, Margolesky J. Features in essential tremor and the development of Parkinson's disease vs. parkinsonism. Neurol Sci 2020;41(11):3249-53 doi: 10.1007/s10072-020-04439-w [published Online First: 20200511].

12. Bugalho P, Salavisa M, Borbinha C, et al. REM sleep behaviour disorder in essential tremor: A polysomnographic study. J Sleep Res 2021;30(2):e13050 doi: 10.1111/jsr.13050 [published Online First: 20200423].

13. Doronina KS, Illarioshkin SN, Doronina OB. [The influence of parasomnia on clinical and functional characteristics of extrapyramidal disorders]. Zh Nevrol Psikhiatr Im S S Korsakova 2021;121(9):13-18 doi: 10.17116/jnevro202112109113.

14. Bugalho P, Meira B, Pinho A, et al. REM sleep behavior disorder and Prodromal Parkinson's Disease in patients with Essential Tremor. Sleep Med X 2024;8:100118 doi: 10.1016/j.sleepx.2024.100118 [published Online First: 20240704].

15. Singh RP, S M, Seshagiri DV, et al. Polysomnographic Evaluation of Sleep Disorders in Essential Tremor and Essential Tremor Plus: A Comparison With Healthy Controls. J Mov Disord 2025;18(1):45-54 doi: 10.14802/jmd.24191 [published Online First: 20241028].

16. Huang HY, Xu F, Zhang D, Luo AL, Xu YM. Prevalence of and risk factors for probable rapid eye movement sleep behavior disorder in Chinese patients with essential tremor. Sleep Med 2025;133:106666 doi: 10.1016/j.sleep.2025.106666 [published Online First: 20250705].
